# Developing a Maternal Health Education and Research Training Program for High School, Pharmacy, and Health Sciences Students

**DOI:** 10.3390/ijerph22071092

**Published:** 2025-07-09

**Authors:** Grace Olorunyomi, Cecilia Torres, Kennedi Norwood, Lashondra Taylor, Jazmyne Jones, Kimberly Pounds, Kehinde Idowu, Dominique Guinn, Denae King, Veronica Ajewole-Mwema, Ivy Poon, Esther Olaleye

**Affiliations:** 1 College of Pharmacy and Health Sciences, Texas Southern University, Houston, TX 77004, USA; grace.olorunyomi@tsu.edu (G.O.); cecilia.torres@tsu.edu (C.T.); kennedi.norwood@tsu.edu (K.N.); jazmyne.jones@tsu.edu (J.J.); kimberly.pounds@tsu.edu (K.P.); kehinde.idowu@tsu.edu (K.I.); veronica.ajewole@tsu.edu (V.A.-M.); 2 Independent Researcher, Houston, TX 77004, USA; lashondrataylor1@gmail.com; 3 College of Education, Texas Southern University, Houston, TX 77004, USA; dominique.guinn@tsu.edu; 4 Barbara Jordan-Mickey Leland School of Public Affairs, Texas Southern University, Houston, TX 77004, USA; denae.king@tsu.edu

**Keywords:** maternal health, maternal education, research training, community engagement

## Abstract

Maternal mortality and morbidity are critical health challenges in the U.S., and building the perinatal workforce is a key to providing high-quality maternal medical care and services. Texas Southern University (TSU), home to a Doctor of Pharmacy program, launched the first Maternal Health Education and Research Training (MHERT) program to educate a cohort of high school, pharmacy, and health sciences students. Aiming to raise awareness of maternal health issues, build research skills, and promote action-based solutions. MHERT integrated online self-paced interactive lessons with hands-on research or community projects. Topics included maternal health epidemiology, causes of morbidity and mortality, research methods, literature reviews, and the development of action plans addressing maternal health challenges. Assessment tools included quizzes, open-ended reflection responses, training surveys, and course evaluations. Running from 3 June to 26 July 2024, the program enrolled 22 students. All participants completed both course components. Course evaluations showed strong and consistent satisfaction with the program, with teaching effectiveness rated at 95% and 96% for mid-program and final evaluations, respectively. MHERT enhanced participants’ understanding of maternal health, improved research skills, and encouraged community engagement and interdisciplinary collaboration. It offers a scalable model to strengthen public health education among high school, pharmacy, and health sciences students.

## 1. Introduction

Maternal mortality and morbidity, defined as complications related to pregnancy, childbirth, and postpartum, are a critical health problem in the United States (U.S.). In 2022, the White House Blueprint for Addressing the Maternal Health Crisis reported that the U.S. has the highest rates of maternal mortality compared to any developed country in the world, despite the advances in medicine and technology [1]. Among all the States in the U.S., Texas is one of the States with the highest maternal mortality rates, with a disturbingly increasing trend from 17.0 deaths per 100,000 live births in 2018 to 24.2 deaths per 100,000 live births in 2020, excluding COVID-19 deaths [2]. This maternal health crisis is particularly devastating for Black or African American (AA) mothers, who are more than three times more likely to die from maternal-related causes nationally [1,3] and two times more likely in Texas than White women [2].

To overcome this maternal health crisis, expanding the perinatal workforce is one of the strategic goals in Texas and nationally [1,2], due to the longstanding shortage of health professionals in primary care and obstetrics providing high-quality maternal care in the U.S. [4]. Additionally, studies have reported the emerging role of community health workers and doulas in increasing access to maternal health services and improving birth outcomes [5,6]. Furthermore, there is increasing attention to expanding the role of community pharmacists in providing perinatal screenings for chronic health conditions [7]. Therefore, producing a pipeline of individuals interested in pursuing healthcare professional careers such as physicians, nurses, pharmacists, and community workers (e.g., community health workers, doulas) is crucial to meeting the increasing needs for maternal care [8].

To meet this emerging need, Texas Southern University (TSU) established the Gulf Coast Collaborative Center for Maternal Health Research, Education, Advanced Training, Community Engagement, and Health Empowerment (MHREACH), funded by the Health Resources and Services Administration (HRSA). The MHREACH program involves two research projects, community engagement activities, and an investigator training and development program for undergraduate, graduate, postdoctoral fellows, and junior faculty. This report describes the development and implementation of our first MHREACH Maternal Health Education and Research Training (MHERT) program for a cohort of high school, pharmacy (PharmDs), and health sciences (B.S./M.S. in Health Administration) students at Texas Southern University (TSU) in the summer of 2024. The goals of the training program are (1) to increase knowledge and awareness of maternal health epidemiology in the U.S.; (2) to enhance research knowledge and skills in exploring maternal health topics; and (3) to support and develop action plans, projects, and research in addressing maternal health problems. These goals aim to achieve the national broad goals of expanding the perinatal workforce.

## 2. Materials and Methods

### 2.1. Curriculum Development

The investigator team (IP, CT, KP, GO) started developing the MHERT program in early 2024, approximately four months before the course target launch month, June 2024. A needs assessment was conducted among faculty, students, and community health workers prior to the development of this course to assess the need for maternal health education, and this helped to guide the development of the curriculum. The MHERT program has two components: (1) an online self-paced course and (2) hands-on research or community project activities. Students must complete the online self-paced course to start the hands-on activities with a mentor. The online course is designed to be delivered through an online independent learning platform called NearPod^®^ [9] to maximize adaptations and replicability for continued education offerings to community health workers and health professionals in the future. Lessons were created in PowerPoint and uploaded to the NearPod platform, and the teaching faculty recorded their lectures on each slide in the lesson. The lessons were self-paced to allow students to learn at their own speed and pause or resume as needed. Each lesson includes the instructional slides and interactive learning activities such as matching pairs, drag-and-drop exercises, multiple choice questions, and fill-in-the-blank knowledge checks to help maintain the student’s attention. The teaching faculty involved a multidisciplinary team of faculty and research staff in the MHREACH program with expertise in pharmacy, epidemiology, public health, and environmental toxicology (IP, CT, KP, GO). An advanced practice pharmacy student reviewed each lesson and provided feedback prior to the implementation of the program.

A syllabus was developed listing the goals of the MHERT program, learning outcomes, teaching faculty, mode of delivery, target learners, expectations, learner requirements, and lesson schedule with objectives. There were eight lessons aimed at providing a general overview of maternal health topics and problems in the U.S. and Texas, common research methods, and a project for students to participate in research related to maternal health, see Table 1.

Upon completion of the online lessons, learners were assigned to a research project with an MHREACH faculty or research staff mentor. A research folder was created in the Box Drive to store and communicate research project assignments. Learners had the opportunity to select whether they preferred an individual or a group project. After the research project assignment, MHREACH research staff host weekly Zoom meetings to check on research progress. To adapt to the knowledge level of learners who might not have prior research experience, we assign research projects that are feasible to be completed in one month’s time. For example, analyzing survey responses that have already been collected, receiving training on how to conduct an interview for research, and learning how to review focus group transcripts to find common themes.

### 2.2. Course Offering

The course was offered from 3 June 2024, to 26 July 2024. Prior to the beginning of the course, program staff (CT, IP) hosted a virtual orientation via Zoom to discuss course expectations and complete agreement forms with students and their parents (for those under 18 years old). A Google Classroom was created to post the syllabus, Nearpod lessons links, and learners’ course progress, and to allow students to ask questions through online chat. Due to the program’s online component, the MHREACH program hired a part-time online instructional expert to guide course design and implementation and provide technical support. High school students received a $250 stipend upon completing the program, supported by the CareSource Bayou Health Foundation.

### 2.3. Participant Recruitment

The MHREACH program staff (CT, IP) created a flyer to advertise the MHERT offering in March 2024. The flyer described the program components, including an 8-week summer research opportunity for high school students that involves an online self-paced learning course, community service hours, and a mentored research project in maternal health. The flyer was emailed to high school counselors and principals in the Greater Houston communities. Eligibility criteria included students entering 11th or 12th grade in high school, with applicants agreeing to commit to at least 8 h per week for an 8-week program. The high school applicants submitted completed applications, a letter of recommendation from a science teacher, and a copy of their high school transcript by mid-April 2024. The program staff (CT, IP, EO) reviewed all received applications and selected the top candidates based on course grades, overall GPA, and their letters of recommendation. Additionally, pharmacy and undergraduate students were recruited from a class announcement by faculty (IP) and through the TSU Leadership, Education, and Advancement in Undergraduate Research Pathways (LEAP) Summer Program.

### 2.4. Assessment Methods

#### 2.4.1. Assessment of Student Performance in Online Lessons

Student assessments were given through the NearPod platform. Quizzes, open-ended responses, and matching games were built into each lesson in NearPod 9.5 software. The program staff (LT) generated weekly reports on students’ work progress, which included the percentage of lesson completion, quiz scores, and responses to open-ended questions. Program staff also developed a shared Excel table that tracked the learner’s progress and categorized each lesson as “completed”, “in progress”, or “not started”. This table was shared weekly on Google Classroom to keep the students informed of their status and encourage continued engagement. A completion grade was defined as a full participation score in each Nearpod lesson and ≥80% correct in the post-lesson quiz. Multiple attempts were allowed, and the highest score attempt was used to track students’ progress.

#### 2.4.2. Pre- and Post-Training Assessments

An online pre- and post-training survey was given to all participants at the start and completion of the program, respectively. Participation in the surveys was voluntary, and each participant completed the informed consent process before participation. The pre-training survey consisted of six open-ended questions designed to gather information on students’ career plans, potential college majors, the rationale for enrolling in the MHERT program, and understanding of current maternal health problems. The post-training survey included five open-ended questions on how the program impacted the participants’ career planning, their understanding of the maternal health crisis, and action steps they would take to address maternal health problems. Texas Southern University’s Institutional Review Board reviewed and approved the survey instruments and study procedures before implementation.

#### 2.4.3. Assessment of the Course

Mid-course and final course evaluations were administered, each comprising 10 Likert scale questions evaluating the quality of the lessons and an open-ended response to provide feedback. Program staff (CT, IP) reviewed the evaluations during the program to guide course modifications. Participation in the course evaluation was voluntary.

## 3. Results

### 3.1. Enrollment and Demographics

A total of 22 students were accepted into the MHERT program, including 10 high school students, 9 pharmacy students, 1 undergraduate pre-health professional student, 1 graduate student in health sciences, and 1 clinical Pharm.D. postdoctoral fellow. Detailed demographics of student participants are shown in Table 2.

### 3.2. Pre- and Post-Training Assessments Results

A total of 15 participants completed the pre-assessment, and 6 completed the post-assessment surveys. In the pre-assessment responses, participants shared that they joined the program due to personal experiences with maternal health challenges, a passion for public health, and aspirations for careers in health-related fields and research. One high school participant wrote:

*“I wanted to apply for this training program as I wanted to gain more background experience in the public health field. While I don’t want to enter the medical field, I plan to major in public health in college because public health and ensuring proper healthcare is something that is important to me, so gaining this experience will help me understand what goes on behind the scenes in this field along with preparing me for the future. Maternal health is also something that I care about because of my familial history of having negative experiences during maternity, so the combination of public health and maternal health provides for this experience to be one of value to me.”*

The post-assessment responses revealed that the program deepened students’ understanding of maternal health topics and equipped them with research and leadership skills to address these issues. When asked about how the program helped towards their career goal, one participant wrote:

*“It has helped me towards my career goal in medicine/research by allowing me to learn a variety of useful information, such as research and presentation skills, maternal health research, and many other valuable skills and topics. I am very much interested in women’s health, and this program has allowed me to dive deeper into maternal health topics, which is wonderful!”*

Another participant shared their research experience:

*“This research training has helped my professional development by educating me on how to properly do in-depth research on any topic and further my understanding.”*

The respondents indicated an interest in volunteering for future community outreaches and expressed a commitment to raising awareness on maternal health problems in their community. When asked about what steps they have taken or plan to take to address maternal health problems after completion of the course, a participant replied:

*“In addition to completing my community project, which involved interviewing Community Health Workers and shedding light on the inequities experienced by mothers in their time of need, I plan to continue my participation with MHREACH and incorporate my knowledge into my education.”*

Another participant wrote:

*“My plan is to continue research on Maternal Health, specifically analyzing hypertension in women, maternal health inequalities, and ways to manage/treat this condition with new focus groups and studies.”*

The participants expressed a desire to be supported further by the MHREACH program through the provision of additional resources and future participation in events, programs, and research related to maternal health and other healthcare fields that can impact the community.

One participant wrote, *“It would be amazing if the MHREACH program could provide me with connections to participate in healthcare policy and legislative advocacy regarding maternal disparities. Along with that, I would also love to continue participating in the research project I was a part of.”*

### 3.3. Mid-Course and Final Course Evaluations

A total of 18 students completed the mid-course evaluation, and 20 completed the final-course evaluation. Both evaluations included eight Likert scale questions, each with response options ranging from “Strongly Agree” to “Strongly Disagree”, along with a question on overall teaching effectiveness, and an open-ended section for additional comments to improve the course. All the responses were on the positive side of the scale for all questions. The overall rating of the course’s teaching effectiveness (0 = least effective, 100 = most effective) averaged 94.67 at mid-course and 95.95 at the final evaluation, suggesting consistent approval of the course’s teaching approach and format throughout the course duration. Detailed information on the mid- and final-course evaluation is shown in Table 3.

At the end of the MHERT training, participants showcased their research and community projects at the TSU LEAP Leadership Summit 2024, a one-day, in-person symposium. Posters were presented by the high schoolers as individuals or as a group to an audience that included Texas Southern University students, staff, faculty, and participants’ parents. The topics for the posters were:

- Analysis of Hypertension in Women: A focus group discussion
- Perspective on Maternal and Child Health: An In-Depth Look at Community Health Professionals’ Experiences
- Thematic Analysis on Maternal Health Education: Maternal and Child Healthcare
- Thematic Analysis on Maternal Healthcare: Stepping towards Heart Care

A presentation of a certificate of completion culminated their training experience. The program will be repeated in summer 2025, with 20 new students expected to enroll, and 6 students from the summer 2024 cohort will return to serve as peer mentors.

## 4. Discussion

The MHERT program represents a new multidisciplinary approach to maternal health education by integrating public health, epidemiology, and pharmacy professionals into the training of high school, pharmacy, and health sciences students to expose learners to the complexities of maternal health topics. Although pharmacists are health professionals serving the community, the pre-pharmacy and pharmacy curriculum is science- and clinical-heavy, with a limited focus on public health and epidemiology in the maternal population. The focus on learning the maternal problems in a public health context fills the gap in the pharmacy curriculum to train a workforce with an understanding of social determinants of health and environmental factors that may affect access to health resources in this special population. The program offered an opportunity for high school students to be mentored in research by a pharmacy faculty and to present their research findings to undergraduate, graduate students, and faculty in the summer research symposium.

Feedback collected through course evaluations was positive, suggesting high satisfaction with the course. Pre-assessment responses highlighted the need for early exposure to maternal health education to foster early engagement in maternal health research in preparation for future interdisciplinary collaboration and healthcare-related careers in addressing maternal health problems. The post-assessment responses emphasized the program’s effectiveness in broadening participants’ perspectives on maternal health issues, encouraging community project involvement, and inspiring long-term engagement in maternal health research and advocacy. The responses emphasized that participants’ interest in maternal health and research extended beyond the course duration. Many participants expressed a desire and willingness to engage further with the program through additional learning opportunities and future research involvement. Some members of the Summer 2024 high school cohort will continue as peer mentors, and several participants have remained in contact with the program. The sustained engagement of participants, coupled with their interest in future public health modules and continued requests for maternal health resources, reflects the overall success and impact of the program.

The MHERT program increased participants’ awareness of maternal health problems, enhanced research skills, encouraged community engagement, and promoted action-based projects. The program’s interdisciplinary format laid a strong foundation for understanding the multifaceted nature of maternal health, preparing students for collaborative problem-solving in their future academic and professional interests. The high level of satisfaction reported by students, along with the effectiveness of the course design, has informed the expansion of maternal health research training at TSU. As a result, the program has become a recurring offering each summer, further institutionalizing maternal health education at TSU.

The asynchronous online mode of teaching allows flexibility in participants’ summer schedules. One potential improvement for this program is to increase student engagement in the NearPod lessons, as shown in our results, where about 10–12% of learners did not agree that the NearPod lessons maintained their attention. Although we had built active learning activities (i.e., reflections, quizzes) into each NearPod activity, each lesson was about one hour, which might be lengthy for high school students. In future offerings, we plan to incorporate group assignments to increase student engagement and discussions among the group.

## 5. Limitations

A limitation of this study is the low response rate for the post-assessment survey. Of the 22 total participants, only 6 completed the post-assessment. This low response was due to several factors: informed consent was required to complete the survey, and not all students provided consent; the survey was only administered to the high school cohort; and it was distributed by email after the completion of the summer program, making it difficult to rally all participants. Although 9 out of 10 high school students provided consent, only 6 completed the survey. To improve response rates in future offerings, post-assessments will be given prior to the final presentations on the day of the symposium, followed by email reminders, and will also be extended to all participants. Another weakness of the program is the lack of a learning model to guide curriculum development. However, the leading developer of this curriculum (IP) has 20+ years of classroom teaching experience, is an experienced NearPod user, and has served as the Chair of Curriculum and Assessment in the Pharm.D. Program. Experiences in classroom management helped to track students’ progress and provided additional support needed to complete the program.

## 6. Conclusions

The success of the MHERT program offers several important implications for public health education and perinatal workforce expansion. Reaching high school students early in their academic endeavors offers exposure to public health-related knowledge that may aid them in deciding their majors in college. The interdisciplinary nature of the program exposes learners to different career disciplines, such as epidemiology, public health, sociology, health policy, and other healthcare professions. Some participants demonstrated interest in maternal and public health in the post-assessment surveys. Additionally, focusing on maternal health epidemiology, a topic that has not been emphasized in our Pharm.D. curriculum, also complements pharmacy students’ training and education to serve the public in the community. The asynchronous online delivery provides a flexible and scalable model for implementation that fits the learner’s summer schedule and supports successful completion of the course. Overall, the inaugural success of this training program has provided many experiences and feedback from the learners to improve upcoming offerings.

## Figures and Tables

**Table 1:** MHREACH course learning outcomes and lesson plans.

I. Course Learning Outcomes		
By the end of the course, learners will be able to: Recognize the critical maternal health problems in the United States. Explain the common causes of maternal health problems. Gain understanding of current research designs commonly used in maternal health studies. Conduct a literature search using a biomedical database. Participate in a research or community project guided by program staff. Create an action plan to address one maternal health problem.		
II. Lesson Plans		
Lesson	Topic	Objectives
1	Plagiarism training	Know the types of plagiarism. Adopt the honor code to avoid plagiarism.
2	Understanding MH epidemiology in the U.S.	Explain the maternal health problems. Understand common maternal health indicators and their utilities. Recognize geographical areas and populations in Texas where maternal health crises are most severe.
3	Causes of MH problems	List the leading causes of pregnancy related death in Texas. Reflect on the causes of MH problems.
4	Policy and Health	Describe how historical public policies have affected geographical residency and impacted health outcomes. Discuss examples of challenges related to accessing healthcare and education in neighborhoods.
5	Factors that contribute to maternal health problems in populations at risk	Describe social determinants of health. Complete an implicit bias assessment and reflection. Explain implicit bias.
6	Human Subject Research	Complete CITI Lessons: Responsible conduct of human subjects research. Understand the IRB application and informed consent.
7	Common Research Methods in Addressing Maternal Health Problems	Describe the types of clinical trial designs. Explain community-based participatory research and its role.
8	How to conduct a literature review and search	Define primary, secondary, and tertiary literature. Utilize the PICO to understand an original intervention study. Identify a maternal health topic and conduct a literature search.
9	Action Steps	Identify goals and actions within the next 3–12 months. Declare an interest in either conducting a research project or being involved in community services about maternal health topics.

**Table 2:** Student demographics (*n* = 22).

Characteristics	Category	Number (%)
Age range (in years, mean age = 21.68)	15–18	10
	19–22	2
	23–26	5
	27+	5
Gender	Female	20 (91%)
	Male	2 (9%)
Race and Ethnicity	Asian	6 (27%)
	Black or African American	13 (59%)
	White	1 (5%)
	Hispanic	2 (9%)
Academic Status	High School Student	10 (46%)
	Undergraduate Student	1 (5%)
	Pharmacy Student	9 (41%)
	Graduate Student	1 (5%)
	Clinical PharmD Postdoctoral Fellow	1 (5%)

**Table 3:** Mid- and final-course evaluation.

Questions	Responses	Mid-Course Evaluation (*n* = 18) Number (%)	Final-Course Evaluation (*n* = 20) Number (%)
1. Each week’s course learning objectives are clearly communicated	Strongly Agree	15 (83%)	14 (70%)
	Agree	2 (11%)	6 (30%)
	Somewhat Agree	1 (6%)	0
2. The instructors speak clearly and audibly when presenting information in NearPod lessons.	Strongly Agree	14 (78%)	13 (65%)
	Agree	4 (22%)	6 (30%)
	Somewhat Agree	0	1 (5%)
3. The NearPod lessons hold my attention when presenting information.	Strongly Agree	11 (61%)	11 (55%)
	Agree	5 (28%)	7 (35%)
	Somewhat Agree	1 (6%)	1 (5%)
	Neither Agree nor Disagree	1 (6%)	1 (5%)
4. The lessons are presented at an appropriate pace.	Strongly Agree	12 (67%)	12 (60%)
	Agree	6 (33%)	8 (40%)
5. The lessons teach me new information.	Strongly Agree	14 (78%)	16 (80%)
	Agree	3 (17%)	4 (20%)
	Somewhat Agree	1 (6%)	0
6. The instructors treat me with respect.	Strongly Agree	14 (78%)	15 (75%)
	Agree	3 (17%)	5 (25%)
	Neither Agree nor Disagree	1 (6%)	0
7. In the NearPod lessons, the instructors explain concepts and ideas by providing examples and relevant applications.	Strongly Agree	15 (83%)	16 (80%)
	Agree	3 (17%)	4 (20%)
8. The discussions and reflection assignments challenge me to think.	Strongly Agree	11 (61%)	14 (70%)
	Agree	6 (33%)	6 (30%)
	Somewhat Agree	1 (6%)	0

## Data Availability

The original contributions presented in this study are included within the article, and raw data supporting the findings of this study will be made available by the corresponding authors upon reasonable request. Further inquiries may be directed to the corresponding authors.

## Abbreviations

The following abbreviations are used in this manuscript:

U.S.	United States
TSU	Texas Southern University
MHREACH	Maternal Health Research, Education, Advanced Training, Community Engagement, and Health Empowerment
HRSA	Health Resources and Services Administration
MHERT	Maternal Health Education and Research Training
MH	Maternal Health
CITI	Collaborative Institutional Training Initiative
GPA	Grade Point Average
LEAP	Leadership, Education, and Advancement in Undergraduate Research Pathways
IP	Ivy Poon
CT	Cecilia Torres
KP	Kimberly Pounds
GO	Grace Olorunyomi
EO	Esther Olaleye
LT	Lashondra Taylor
